# Persistent Symptoms Post-COVID-19: An Observational Study at King Abdulaziz Medical City, Jeddah, Saudi Arabia

**DOI:** 10.7759/cureus.24343

**Published:** 2022-04-21

**Authors:** Maryam A Jabali, Ahmad S Alsabban, Lujain M Bahakeem, Mohammad A Zwawy, Abdulaziz T Bagasi, Hessan T Bagasi, Taghreed A Aldosary

**Affiliations:** 1 Family Medicine, Ministry of the National Guard-Health Affairs, King Abdulaziz Medical City, Jeddah, SAU; 2 Medicine, Hera General Hospital, Makkah, SAU; 3 Medical Sciences - Oral Biology, Ministry of the National Guard-Health Affairs, King Abdulaziz Medical City, Jeddah, SAU

**Keywords:** post covid-19, persistent symptoms, persistent covid-19, sars-cov, covid-19

## Abstract

Background

As the prevalence of COVID-19 recovery cases increased, patients started to notice new symptoms after being cured of the acute infection. We aimed to study the type of persistent symptoms post-COVID-19 infection, their prevalence, and factors that play a role in developing the post-COVID-19 symptoms among COVID-19 patients at King Abdulaziz Medical City, Jeddah, Saudi Arabia.

Methods

A cross-sectional study was conducted at King Abdulaziz Medical City, Jeddah, Saudi Arabia, from the period of September 2021 to December 2021. Participants were contacted via a phone interview. Statistical analysis was performed using IBM SPSS Statistics, and p-values of ≤0.05 were considered significant.

Results

A total of 327 participants completed the study, of which 169 (51.7%) were male. Nearly half of the patients, 161 (49.09%), had persistent symptoms. The most common symptoms were loss of smell, loss of taste, cough, and fatigue (22.6%, 19.2%, 11.6%, and 9.1% respectively). They were followed by an equal percentage of shortness of breath, headache, and hair loss (7.3%). Gender was found to be significant in loss of smell, loss of taste, and hair loss, with p-values of 0.016, 0.018, and <0.001, respectively.

Conclusion

A large proportion of patients with COVID-19 developed persistent symptoms. The most common symptoms were loss of smell and taste, cough, and fatigue. Some factors played a role in acquiring post-COVID-19 symptoms, including gender and place of treatment. Gender was significantly associated with hair loss. Follow-up after recovery is required to maintain individual well-being.

## Introduction

By the end of 2019, a cluster of pneumonia of unknown origin cases has occurred in Wuhan City, China, as discovered to be caused by coronavirus disease 2019 (COVID-19), which has spread rapidly to cause a worldwide pandemic [1-3]. In March 2020, The Kingdom of Saudi Arabia (KSA) declared the first case of COVID-19 [4].

Clinical characteristics of COVID-19 showed a variety of symptoms ranging from mild to severe symptoms as reported by the CDC [5]. A study conducted in Saudi Arabia showed that particularly older age and those with underlying comorbid diseases such as cardiovascular disease, diabetes, chronic respiratory disease, and cancer developed severe symptoms that required longer hospital admission or intensive care unit (ICU) admission [4]. Previous studies showed that the most common symptoms were fever, cough, dyspnea, fatigue, and myalgia, in addition to alteration in smell or taste, which was described by 64.4% of COVID-19 patients [3,6,7]. In Saudi Arabia, the most prevalent symptoms were fever, cough, sore throat, and shortness of breath [4,8].

Persistent symptoms were defined as ongoing or new symptoms that developed post-recovery from acute COVID-19 infection and were persistent for at least two months [9]. Many patients reported symptoms of COVID-19 after recovery from the acute phase of infection, and most of them experienced fatigue and dyspnea as persistent symptoms within one to three months after being discharged from the hospital [6,10-12], with no statistical difference between patients admitted to ICU and non-ICU admission [10]. Age of 40 to 60 years and abnormal auscultation at symptoms onset were markedly found to play a role in persisting symptoms [6]. The majority of patients with mild illness who were managed in an outpatient setting reported ongoing symptoms after diagnosis, mainly fatigue, cough, and headache [13].

As the prevalence of COVID-19 recovery cases increased, patients started to notice new symptoms after being cured of the acute infection. In this article, we aim to study the type of persistent symptoms post-COVID-19 infection, their prevalence, and factors that play a role in developing the post-COVID-19 symptoms in COVID-19 patients at King Abdulaziz Medical City, Jeddah, Saudi Arabia.

## Materials and methods

Study design and setting

A cross-sectional study was conducted at King Abdulaziz Medical City from the period of September 2021 to December 2021.

Sampling

The total population of COVID-19 patients at King Abdulaziz Medical City at the time of the study was 2,151. The calculated sample size was 327 with a confidence interval of 95% and a 5% margin of error and the calculations were performed using the Raosoft sample size calculator (http://www.raosoft.com/samplesize.html).

All adult patients aged 18 years old and above who were previously diagnosed with COVID-19 through nasopharyngeal swab test via polymerase chain reaction (PCR) were included. All those who did not confirm their COVID-19 diagnosis via PCR test, those who did not answer the phone three times, 10 minutes apart, those who could not communicate for any other reason, i.e., deafness, mental retardation, and inability to speak, those who died, and those who refused to participate were excluded.

Data collection

Data were collected from the medical record department, and participants were contacted via a phone interview at least six months post-COVID-19 infection. A pilot study was conducted, and the questionnaire was reviewed by two bilingual specialized consultants that are experts in the field. Reliability was assessed using Cronbach’s alpha 0.661.

The questionnaire was composed of four parts. The first part includes demographics such as age, gender, height, weight, date of diagnosis (data of COVID-19 test result), smoking status, the type used (cigarette, e-cigarette, or Hookah) and duration of smoking by years, activity on a weekly basis before COVID-19 infection, and the duration of the activity. The second part consists of questions regarding the patient’s symptoms before, during, and after COVID-19. The third part is related to comorbidities such as diabetes, lung disease, cancer, heart disease, kidney disease, liver disease, anxiety, low mood, high blood pressure, irritable bowel syndrome (IBS), obesity, joint pain/rheumatoid arthritis, and cerebrovascular accident before COVID-19. The last part related to treatment methods during COVID-19 infection, follow-up by a medical practitioner (telephone consultation), clinic visit, emergency room (ER) assessment without hospitalization, admission to a regular or intensive care unit, use of oxygen at home before, during, and after infection, and vaccination details (brand, numbers of doses, time of administration before or after the infection).

Statistical analysis

Data were entered into Microsoft Office Excel version 2022, and statistical analysis was conducted using IBM SPSS Statistics Version 23 (IBM Corp., Armonk, NY, USA) and visually presented using GraphPad Prism version 8 (GraphPad Software, Inc., San Diego, CA, USA). Descriptive data were represented as mean, standard deviation (SD), or minimum to maximum for continuous values, and as number (%) for categorical values. The chi-square tests and an independent t-test were used to detect significant differences between groups for categorical and continuous data, respectively. P-values of ≤0.05 were considered significant.

Ethical considerations

Institutional Review Board approval was obtained from King Abdullah Medical Research Center. The participant's verbal consent was obtained. The privacy and confidentiality of the participants were acknowledged.

## Results

A total of 327 participants completed the study, of whom 169 (51.7%) were male. Their age ranged between 18 and 94 years, with a mean age of 46.83 (SD: 16.5) years (Table 1).

**Table 1:** Demographic characteristics

Demographics	N=327 (%)
Age (years)	
18-34	89 (27.2)
35-49	103 (31.5)
50 and older	135 (41.3)
p-Value	0.846
Gender	
Male	169 (51.7)
Female	158 (48.3)
p-Value	0.249
BMI	
Underweight	7(2.1)
Normal	70 (21.4)
Overweight	113 (34.6)
Obese	136 ( 41.6)
p-Value	0.642
Smoking	
Yes	61 (18.7)
No	266 (81.3)
p-Value	0.158
Before the corona infection did you do any activity on a weekly basis	
Yes	146 (44.6)
No	181 (55.4)
p-Value	0.255

**Figure 1:**
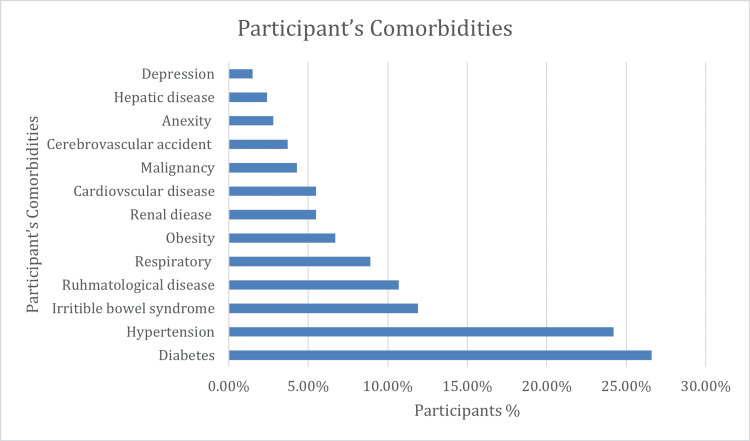
Participants' comorbidities

Regarding comorbidities, 87(26.6%) of the patient had diabetes, 79(24.2%) had hypertension, 29(8.9%) had a respiratory disease, 39(11.9%) had IBS, 35 (10.7%) had a rheumatological disease, and 22 (6.7%) had obesity (Figure 1).

Nearly half of the patients, 161 (49.09%), had persistent symptoms, with no significant relation to age, smoking status, BMI, physical activity, and presence of comorbidities. Gender was found to be significant in loss of smell, loss of taste, and hair loss, with p-values of 0.016, 0.018, and <0.001, respectively.

**Figure 2:**
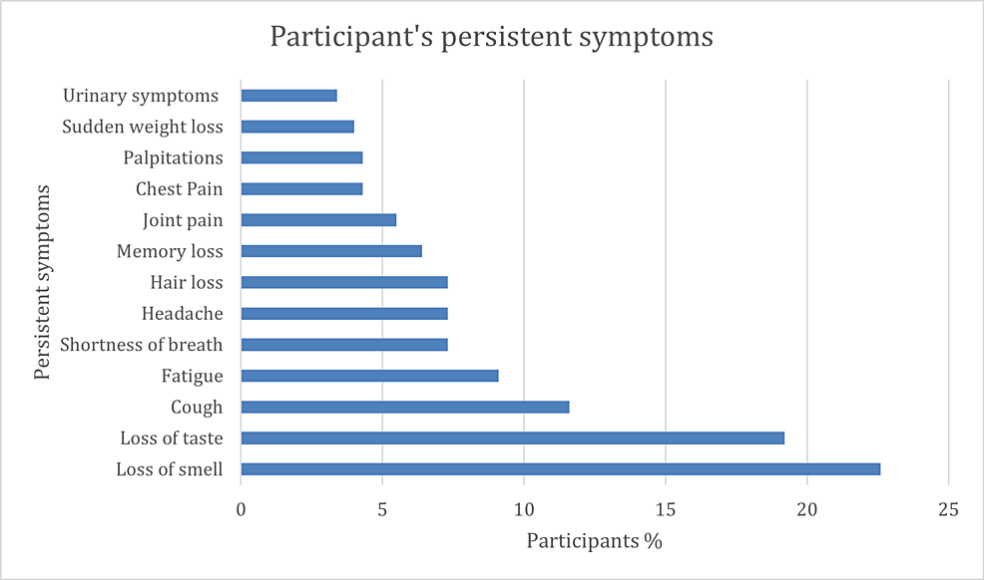
Participants' persistent symptoms

The most common symptoms were loss of smell, loss of taste, cough, and fatigue (22.6%, 19.2%, 11.6%, and 9.1%, respectively), followed by an equal percentage of shortness of breath, headache, and hair loss (7.3%) (Figure 2).

Overall, 36% visited the ER, 31.8% required hospital admission, and 7.6% were admitted to ICU. The remaining were instructed to get isolated at home and were conservatively treated. Admission to the hospital either to a regular ward or ICU was found to be a significant factor in developing persistent symptoms, with p-values of 0.01 and 0.05, respectively. Patients who underwent ER assessment without hospitalization were found to have significant p-values of 0.015 and 0.020 in relation to fatigue and hair loss, respectively.

During the course of the disease, 20.8% required oxygen, and this was statistically significant in acquiring persistent symptoms, with a p-value of 0.020.

In assessing the vaccination status of our contributors, only 2.4% received at least one dose of the vaccine before getting infected.

## Discussion

This observational study aimed to assess the type of persistent symptoms post COVID-19 infection, their prevalence, and factors that play a role in developing the post-COVID-19 symptoms among COVID-19 patients at King Abdulaziz Medical City, Jeddah, Saudi Arabia.

Around half of our patients reported at least one persistent symptom. This is parallel to studies conducted in Saudi Arabia and Bangladesh that showed that approximately half of the patients had persistent symptoms [14-16].

On the other hand, studies found that 76% to 91% had at least one persistent symptom, and this difference in reported percentages can be attributed to the difference in sample size, patients demographics, and severity of the disease, and to the factor that patients who had symptoms before acute COVID-19 illness were not excluded in their studies [11,17,18].

There were notable cases of loss of smell, loss of taste, cough, and fatigue. In accordance with a systematic review that stated that both loss of taste and smell were prevalent persistent symptoms [19]. Several authors reported fatigue as the most common symptom [13-15,18,20-23].

A large cohort study showed that the female gender is one of the potential risk factors for developing post-COVID-19 features [14,18]. This can be clarified by the difference in immunological response between males and females, psychosocial stress, and the assumption that females are more concerned about their health [24,25]. This is consistent with our findings, which showed that loss of smell, loss of taste, and hair loss were significantly higher in females than males. Hair loss was also matched with a result of a study by Garrigues et al. in 2020 [10,16]. On the contrary, there were authors who found no effect of gender on persistent symptoms [15,20].

We found that age, obesity, and pre-existing comorbidities were not associated with developing persistent symptoms. Similarly, Xiong et al. and Moreno-Pérez et al. supported our findings [16,26], although Abdelrahman et al. and Tenforde et al. both found a relation between age and persistent symptoms [13,20]. Evidence revealed that smokers are more vulnerable to severe disease [27,28], although we did not find an association with persistent symptoms.

Our study found a significant difference between outpatient and inpatient persistent symptoms, which is in agreement with a study conducted in Saudi Arabia that showed significant differences depending on the place of treatment [29]. Halpin et al. concluded that patients admitted to ICU had a higher percentage of symptoms [12], whereas Garout et al. found no relation between the severity of the disease and persistent symptoms [15].

Hair loss was significant in patients treated in the ER and this may be related to stress post ER discharge [10].

There are few limitations to the study. The respondents were from a single center and we could not determine the effect of vaccination as most of our patients received the first dose of vaccine after they got infected.

## Conclusions

A large proportion of patients with COVID-19 developed persistent symptoms post-infection. The most common symptoms were loss of smell and taste, cough, and fatigue. Some factors played a role in acquiring post-COVID-19 symptoms, including gender and place of treatment. Gender was significantly associated with hair loss. Follow-up after recovery is required to maintain individual well-being.
